# Refining Protein Subcellular Localization

**DOI:** 10.1371/journal.pcbi.0010066

**Published:** 2005-11-25

**Authors:** Michelle S Scott, Sara J Calafell, David Y Thomas, Michael T Hallett

**Affiliations:** 1 McGill Center for Bioinformatics, McGill University, Montreal, Quebec, Canada; 2 Biochemistry Department, Faculty of Medicine, McGill University, Montreal, Quebec, Canada; Technical University of Denmark, Denmark

## Abstract

The study of protein subcellular localization is important to elucidate protein function. Even in well-studied organisms such as yeast, experimental methods have not been able to provide a full coverage of localization. The development of bioinformatic predictors of localization can bridge this gap. We have created a Bayesian network predictor called PSLT2 that considers diverse protein characteristics, including the combinatorial presence of InterPro motifs and protein interaction data. We compared the localization predictions of PSLT2 to high-throughput experimental localization datasets. Disagreements between these methods generally involve proteins that transit through or reside in the secretory pathway. We used our multi-compartmental predictions to refine the localization annotations of yeast proteins primarily by distinguishing between soluble lumenal proteins and soluble proteins peripherally associated with organelles. To our knowledge, this is the first tool to provide this functionality. We used these sub-compartmental predictions to characterize cellular processes on an organellar scale. The integration of diverse protein characteristics and protein interaction data in an appropriate setting can lead to high-quality detailed localization annotations for whole proteomes. This type of resource is instrumental in developing models of whole organelles that provide insight into the extent of interaction and communication between organelles and help define organellar functionality.

## Synopsis

Eukaryotic cells are divided into various morphologically and functionally distinct compartments. Proteins must be targeted to the appropriate compartment to ensure proper function. Understanding protein subcellular localization is important to help understand not only the function of individual proteins but also the organization of the cell as a whole. Bioinformatic predictors of localization can provide such information quickly for large numbers of proteins. The authors of this paper have created a localization predictor called PSLT2 that considers the combinatorial presence of protein motifs and domains as well as protein interactions in yeast proteins. PSLT2 can predict the localization of all yeast proteins to nine different compartments: the endoplasmic reticulum, Golgi apparatus, cytosol, nucleus, peroxisome, plasma membrane, lysosome, mitochondrion, and extracellular space. The authors also investigated how to identify and predict proteins that localize to more than one compartment. They compared the localization predictions of PSLT2 to those determined through high-throughput tagging and microscopy experiments for yeast proteins. Disagreements between these methods generally involve proteins that transit through or reside in the secretory pathway.

## Introduction

Subcellular localizations determine the environments in which proteins operate. As such, subcellular localization influences protein function by controlling access to and availability of all types of molecular interaction partners. Thus, knowledge of protein localization often plays a significant role in characterizing the cellular function of hypothetical and newly discovered proteins. There are several research endeavours that aim to localize whole proteomes by using high-throughput approaches [1–3]. These large datasets provide important information about protein function, and more generally global cellular processes. However, they currently do not achieve 100% coverage of proteomes, and the methodology used can in some cases cause mislocalization of subsets of proteins [4,5]. Complementary methods are necessary to address these problems.

Many efforts have focused on the creation of bioinformatic predictors of localization via different machine learning methods, and using various protein characteristics (reviewed in [6]). Available predictors can be grouped into four general classes based on the protein characteristics considered: amino acid composition and order-based predictors [7,8], sorting signal predictors [9,10], homology-based predictors [11,12], and hybrid methods that use several sources of information to predict localization [5,13,14]. Existing predictors have shortcomings, which can include low coverage, a small number of compartments considered, low predictive accuracy, and misannotation of several classes of proteins that include multi-compartmental proteins, proteins at the boundary of two organelles, and transmembrane proteins.

To address some of these issues, we have created a localization predictor for yeast proteins called PSLT2 (Protein Subcellular Localization Tool 2) that integrates the presence of motifs, domains, and targeting sequences with protein–protein interaction data. We have previously shown that the integration of various motif, domain, and targeting signal information into a probabilistic framework allows for accurate prediction of mammalian protein subcellular localization [5]. PSLT2 is based on similar methodology but uses additional data available for yeast proteins. PSLT2 achieves a prediction accuracy of at least 72% and a coverage of 100% of the yeast proteome. A comparison of PSLT2 predictions to experimentally tagged high-throughput datasets reveals that most disagreements occur when predicting the localization of proteins belonging to the secretory pathway. Such annotation errors can be identified by combining different experimental and bioinformatic methods. Additionally, PSLT2 predictions were used to study the *sub-compartmental* localization of all yeast proteins, allowing for more specific annotations than presently available. In particular, we investigate how to distinguish between lumenal proteins of organelles and proteins that are associated with these organelles on their cytosolic side. Such distinctions are not widely available in public databases. In fact, many proteins annotated as being inside specific organelles are actually cytosolic proteins that are peripherally associated with the organelle through interactions with organellar membrane proteins or lipids. These sub-compartmental predictions should be extremely useful for the biological community, as they represent a new tool to study cellular processes on an organellar scale. These refined predictions can in turn be used to investigate the extent of interaction and communication between organelles and help define organellar functionality.

## Results

### Components of the PSLT2 Bayesian Network

**Figure 1:**
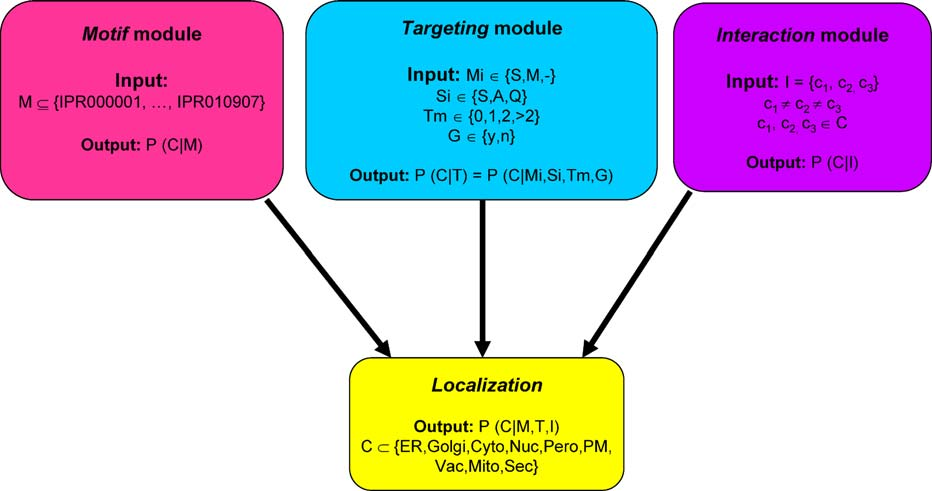
Structure of the PSLT2 Bayesian Network The PSLT2 predictor is composed of three independent modules that can predict localization individually or in combination: the motif, targeting, and interaction modules. Each module can be characterized by the protein information used as input and the localization probabilities (for all compartments [C]) that are generated as output. The motif module accepts combinations of InterPro motifs (M) as input. The targeting module considers the presence of mitochondrial targeting signals (Mi), signal peptides/anchors (Si; S, signal peptide; A, signal anchor; Q, neither), GPI anchors (G), and the number of transmembrane domains (Tm) to predict localization. The interaction module considers the three compartments to which are localized the largest number of interactions partners (see Materials and Methods for more details). The full network (illustrated as the localization module) takes into account the output of all three modules to predict the probability of localization to all compartments (C).

Different types of protein characteristics can be used in the prediction of subcellular localization, including the presence of a wide variety of motifs, domains, and targeting sequences that can be identified from the primary sequence. Bayesian networks provide an excellent tool for the integration of such diverse information because they can generate probabilistic models that are well suited to capture the combinatorial aspect of the data [15]. The full PSLT2 Bayesian network (illustrated in Figure 1) is composed of three independent modules (each capable of predicting localization on its own) whose predictions are combined using a naïve Bayes net localization predictor. The *motif* module is trained to predict localization based on the combinatorial presence of InterPro motifs [16] in proteins. The *targeting* module uses targeting signals such as signal peptides/anchors as predicted by SignalP [9], mitochondrial targeting peptides predicted by TargetP [10], glycosyl-phosphatidylinositol (GPI) anchors predicted by DGPI (http://129.194.185.165/dgpi/index_en.html), and the presence of transmembrane domains predicted by TMHMM [17]. Finally, the *interaction* module is trained on protein–protein interaction data from the CORE set of the Database of Interacting Proteins [18]. The modules are described in greater detail in the Materials and Methods section.

Information used within the targeting module is available for all yeast proteins. However, not all types of information are available for all proteins in the motif and interaction modules. We term a protein as uninformative if it lacks both motif and interaction information. Otherwise, we say it is *informative* (see Materials and Methods). 83% of yeast proteins are informative.

### Statistical Tests of Accuracy

All possible combinations of the three modules can be used to predict localization. To evaluate the contribution of each module, we tested each combination of modules using a 10-fold cross-validation approach. As shown in Table 1, as more information is used by the predictor, its accuracy and coverage increase, suggesting that the different modules provide some complementary information. On one hand, the interaction module alone provides a high prediction accuracy (the highest achieved for a single module) but low coverage. This can be explained by the fact that protein–protein interaction information is a good indicator of localization (proteins must be in close proximity to interact) but protein interaction datasets have high false-negative rates [19]. On the other hand, the targeting module allows 100% coverage (as all proteins can be scanned for the presence of the targeting motifs) but achieves lower predictive accuracy (as these motifs are less informative because they alone are insufficient to distinguish between all compartments). Because the full network achieves the highest accuracy and coverage, PSLT2 is constructed using all three modules.

**Table 1:**
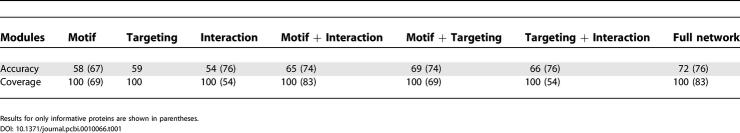
Ten-Fold Cross-Validation Test Accuracy and Coverage for Every Module Combination

Table 2 shows the positive predictive values and sensitivity for all compartments considered, calculated using a 10-fold cross-validation test. The results are shown for all proteins and for only informative proteins. The numbers in parentheses show the results of the second-best test, in which the predictor is allowed to predict the two most likely compartments and a prediction is considered a success if at least one of the two predictions is correct. The second-best test is biologically relevant since many multi-compartmental proteins are not annotated as such in current public databases. The overall prediction accuracy is 72% for all yeast proteins, 76% for the 83% of yeast proteins that are informative, and the second-best test accuracy is above 85% for all yeast proteins. In general, proteins in the nucleus, mitochondria, and secretory pathway organelles are better predicted than proteins located elsewhere in the cell. This is particularly interesting in the case of organelles of the secretory pathway, since available predictors generally either group these organelles into one multi-organelle compartment (thus providing a very unrefined prediction), or they simply achieve low prediction accuracy for these organelles. Predictions for all yeast proteins are available in Table S1.

**Table 2:**
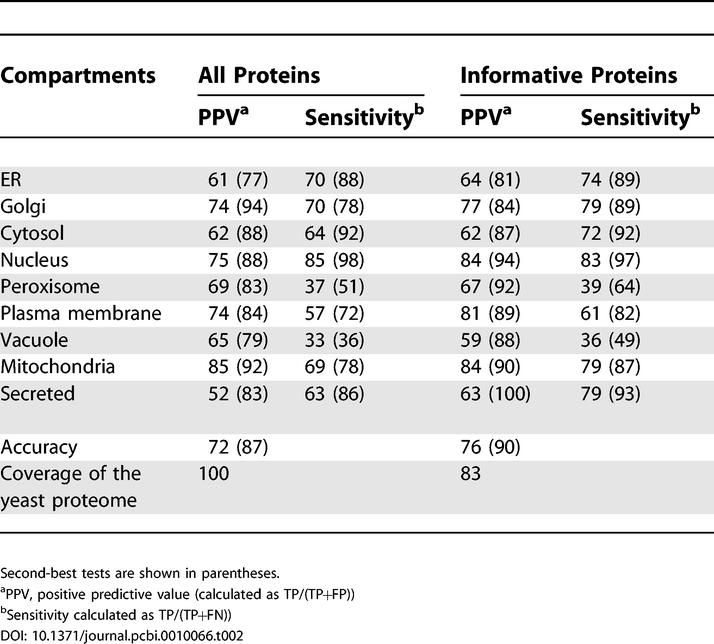
Ten-Fold Cross-Validation Test Results of the Full PSLT2 Localization Predictor

### Proteome-Wide Multi-Compartmental Prediction

PSLT2 can be used to predict the localization of all proteins in the cell. Because it generates localization likelihoods for all compartments, it can also be used to evaluate whether a protein is present in more than one organelle. To do so, proteins for which the localization likelihood of the second highest scoring compartment is above a certain threshold are predicted to be present in both high-scoring compartments. Our previous investigations [5] of this threshold indicate optimal prediction accuracy when the likelihood of the second highest scoring compartment is greater than half of the likelihood of the highest scoring compartment.

Table 3 shows the distribution of proteins in the different compartment pairs. Proteins on the diagonal of Table 3 are predicted to be in only one compartment. Four general classes of multi-compartmental predictions can be identified.

**Table 3:**
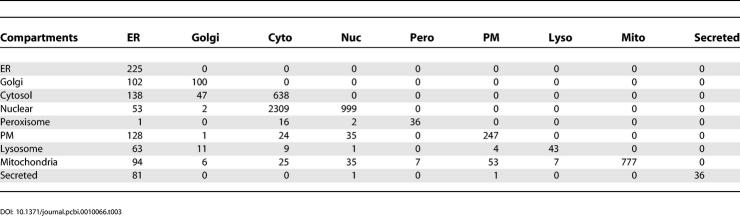
Number of Yeast Proteins Predicted in Every Compartment Pair

The first class contains proteins that shuttle between compartments. A large set of known examples of this class are in the nuclear–cytosolic protein group, many of which are well predicted by PSLT2, including kinases, transcription factors, and proteins that bind RNA (for example, HXK2, CAD1, RIO2, GLE1, PBP1). Another such group of proteins shuttle between the endoplasmic reticulum (ER) and the Golgi, including proteins involved in cargo transport between these two organelles (for example, SEC31, SEC24, ERV14).

The second class of multi-compartmental proteins involves proteins localized at the boundary of organelles. This class includes cytosolic proteins that are associated with the ER (for example, many proteasomal proteins, CDC48, and an ER-associated glutathione GTT1), cytosolic proteins associated with the Golgi apparatus (for example, SEC14, IMH1), as well as membrane proteins of certain organelles that can interact with components of the cytosol (DPM1 in the ER membrane) and nuclear pore complex proteins predicted to be nuclear and cytosolic (for example, NUP84, NUP157).

The third class of predicted compartment pairs involves intermediate compartments through which some proteins transit before reaching their final destination (for example, the ER-plasma membrane and ER-secreted groups including PDR12, DAL5, and some soluble cell wall (SCW) proteins.

The last class of predicted multi-compartmental proteins involves compartment pairs that are less likely to share proteins. The largest sets of such compartment pairs predicted by PSLT2 are the mitochondria–ER group and the mitochondria–plasma membrane group. It is interesting to note that the ER and mitochondria share several proteins in mammalian cells, including components of the apoptosis machinery such as Bcl2 and Bak [20]. Further studies will be necessary to determine the biological significance of the large number of proteins predicted to be localized to both the ER and mitochondrion in yeast, including a possible role in calcium signalling.

### Comparison with High-Throughput Tagged Datasets

Several large-scale high-throughput protein localization experiments have been conducted recently [1–3,21]. The datasets from these screens have substantially increased the number of proteins of known localization. However, since such efforts involve tagging proteins in order to allow their visualization by microscopy, these datasets likely contain a non-negligible number of incorrect localization annotations. For example, N-terminal tagging of proteins has the potential to disrupt signal peptides, and mitochondrial targeting peptides and C-terminal protein tags can mask motifs such as the HDEL and SKL signals used, respectively, for retention and targeting to the ER and peroxisome. Internal tags, although possibly less disruptive for many localization targeting signals, could destabilize the protein, interfere with proper folding, and, in some cases, cause mislocalization.

In order to assess how well PSLT2 predictions agree with localization annotations generated by high-throughput studies and to determine where the disagreements occur, we chose to use the publicly available protein localization data from TRIPLES (http://ygac.med.yale.edu/triples/default.htm) and YeastGFP (http://yeastgfp.ucsf.edu/) due to the high coverage afforded by these datasets. The YeastGFP dataset consists of 4,156 yeast proteins whose localization was established by visualization of chromosomally tagged C-terminal GFP fusion proteins [2]. The TRIPLES dataset was derived by immunolocalization of 2,744 randomly or C-terminally tagged yeast proteins [1].

**Figure 2:**
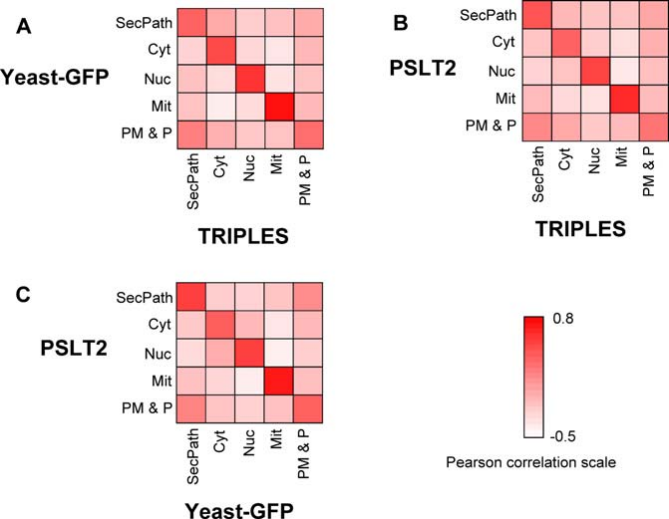
Comparison between PSLT2, TRIPLES, and YeastGFP Datasets Panels A through C represent an illustration of the Pearson correlation for the probability of localization between all compartment pairs for each pair of datasets (see Materials and Methods for details). SecPath, secretory pathway (ER and Golgi); Cyt, cytosol; Nuc, nucleus; Mit, mitochondrial or peroxisomal; PM & P, plasma membrane and periphery (including secreted and vacuolar proteins).

The results of the comparison are shown in Figure 2. Not all compartments were used in the experimental high-throughput localization studies, so we grouped the nine compartments predicted by PSLT2 into five mega-compartments: secretory pathway (abbreviated SecPath, it encompasses ER and Golgi proteins), cytosol (Cyt), nucleus (Nuc), mitochondria/peroxisome (Mit), and plasma membrane and periphery (PM & Periphery, it contains vacuolar proteins, plasma membrane proteins, and secreted proteins). It should be noted that the TRIPLES and YeastGFP datasets use the “cytoplasmic” annotation (which is defined by the Gene Ontology as “all of the contents of a cell excluding the plasma membrane and nucleus, but including other subcellular structures”), whereas PSLT2 uses “cytosolic”, which provides much greater specificity as no organellar proteins are part of this group. As a consequence, proteins in the ER, Golgi, vacuole, mitochondrion, and peroxisome can be classified as cytoplasmic (and some are, especially in the case of some organelles), resulting in confusing and very vague annotation. This is a widespread problem that affects large databases as well as most previous localization predictors whose training sets use cytoplasmic annotations to mean cytosolic localization.

To quantify the degree of similarity between the three datasets, we calculated the Pearson correlation coefficients for localization probability for all pairs of compartments and datasets, over all proteins considered. If the datasets strongly agree, we would see very high Pearson correlation between same compartment pairs, and low Pearson correlation between different compartment pairs (and thus very dark diagonal and very light off-diagonal squares in Figure 2). As shown in Figure 2, there is a much higher Pearson correlation, and thus general better agreement, between all datasets for all same compartment pairs than for different compartment pairs, except in the case of the plasma membrane and periphery group. In general, there is low Pearson correlation between the mitochondria–cytosol and mitochondria–nucleus groups between all datasets, indicating that disagreements do not occur between these compartment pairs. However, in general, much higher Pearson correlation coefficient values are observed involving the secretory pathway, and plasma membrane and periphery groups with other compartments. This indicates that disagreements between datasets involve mostly proteins annotated as being in the secretory pathway, or the plasma membrane and periphery mega-compartments in at least one of the datasets.

We examined general classes of proteins that contain many members known to transit through the secretory pathway or localize in its compartments as well as other groups of proteins whose localization is not agreed upon by the three methods. Table 4 summarizes this analysis and helps explain the discrepancies. In general, in all three datasets, many proteins containing signal peptides as predicted by SignalP [9] are present in the secretory pathway. The situation is similar in the case of the plasma membrane and periphery group for the YeastGFP annotations and PSLT2 predictions, but the TRIPLES dataset annotates more signal-peptide containing proteins as being cytoplasmic rather than in the plasma membrane and periphery group. The high numbers of proteins containing a signal peptide and annotated as being in the mitochondria group is probably in part due to failure of SignalP to discriminate between signal peptides and mitochondrial targeting peptides.

**Table 4:**
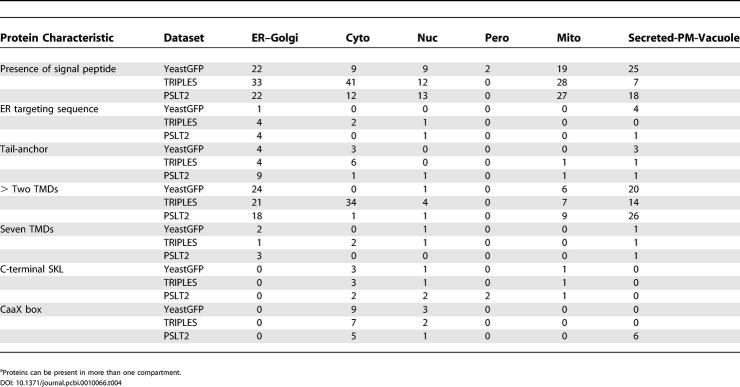
Comparison of Localization Annotations between PSLT2, YeastGFP, and TRIPLES Datasets for Different Classes of Proteins^a^

Only five proteins containing the C-terminal ER retention motif HDEL are present in the three datasets. PSLT2 and the TRIPLES dataset annotated four of them as being in the secretory pathway group. The YeastGFP dataset annotates four of them as being in the plasma membrane and periphery group. This could be due to the C-terminal GFP tag that might be masking the HDEL signal.

Nine of the 56 tail-anchored proteins known to exist in yeast cells [22] are present in the three datasets. All nine of these proteins are predicted by PSLT2 to be in the secretory pathway. The YeastGFP and TRIPLES datasets annotate four of the known tail-anchored proteins as being in the secretory pathway.

Most of the multi-spanning membrane proteins are annotated by the YeastGFP and PSLT2 datasets as being either in the secretory pathway or in the periphery of the cell membrane, whereas a large number of these proteins are annotated by the TRIPLES dataset as being cytoplasmic.

We also looked at the distribution of C-terminal SKL motif–containing proteins, which are expected to localize to the peroxisome. There are four such proteins in the dataset. Two are predicted as being peroxisomal by PSLT2 (one of which is also predicted to be mitochondrial). None are annotated as peroxisomal in the YeastGFP and TRIPLES datasets.

Nine proteins in the dataset contain a C-terminal CaaX-box motif (which is a targeting signal that specifies prenylation) as defined in PROSITE (motif ID: PS00294). These motifs are believed to be used to anchor proteins in diverse cellular membranes including the plasma membrane [23]. Six of these proteins are predicted to be in the plasma membrane and periphery group by PSLT2. The YeastGFP and TRIPLES datasets annotate these proteins as being elsewhere in the cell.

### Comparison with Previous Methods

To accurately and fairly compare predictors, one should ideally use an independent set that does not contain any sequences used to train the predictors. Unfortunately, such a dataset does not exist for yeast proteins, as most predictors use all the UniProt/SwissProt [24] annotations available. Annotations resulting from high-throughput datasets cannot presently be considered as gold standards as they suffer from some biases caused by the experimental procedure, as discussed in the previous section. Related fungi species could be used in an independent test on PSLT2, but many previous methods use these proteins in their training sets. Furthermore, interaction datasets for fungal species other than *Saccharomyces cerevisiae* contain very few interactions. As a consequence, in Table 5, we compare PSLT2 features and accuracy to those reported for previous methods, instead of performing an independent test. We considered publicly available predictors for which the prediction accuracy is reported for all compartments. These predictors include SubLoc [8] and PLOC [25], both of which are based on support vector machines and consider amino acid composition (SubLoc and PLOC), amino acid pair, and gapped amino acid pair compositions (PLOC) in eukaryotic proteins. We also consider the non-plant eukaryotic version of LOCtree [26] and the Proteome Analyst predictor trained on fungal sequences [12]. LOCtree is a hierarchical system that combines support vector machines and other prediction methods based on homology and keywords. The Proteome Analyst predictor uses homology information and SwissProt annotations to predict localization. As shown in Table 5, not all predictors consider transmembrane proteins, and PSLT2 is the only predictor to attempt to predict multi-compartmental proteins in a systematic way. The predictors vary in the number of compartments considered and the accuracy achieved for each compartment. PSLT2 is particularly successful for proteins localized to secretory pathway organelles and mitochondria, which are notoriously difficult to predict. In general, these five predictors achieve a similar overall prediction accuracy and coverage. However, care must be taken when choosing a predictor, as not all predictors consider all compartments and most do not predict all proteins (note that PSLT2 can predict localization for all proteins but achieves a slightly lower accuracy in this case; see Table 2). In general, predictors that consider homology achieve high prediction accuracy for proteins with close homologues but much lower accuracy for proteins that do not have close homologues. Methods such as PSLT2 are complementary to the homology-based methods and allow accurate predictions in the absence of well-annotated homologues.

**Table 5:**
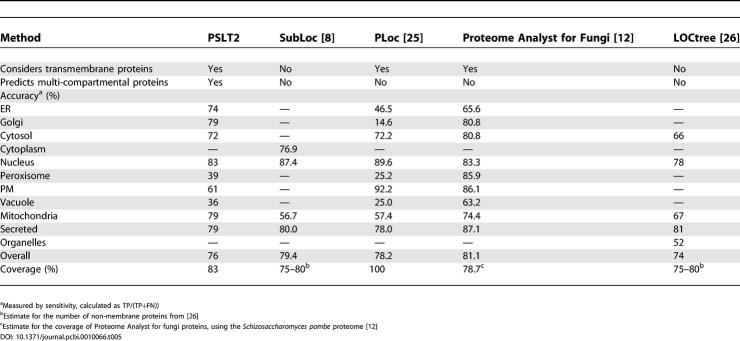
Comparison with Previous Methods

### Sub-Compartmental Prediction

In searching through several of the large protein databases, we have observed that many soluble proteins annotated as being inside the ER are actually peripherally associated with the ER on the cytosolic side [27]. This is a widespread problem that also affects other organelles. Such misleading annotations likely result from the difficulty of distinguishing experimentally between these precise localizations. Methods to address this problem are urgently needed because of the large number of proteins it affects and the numerous research groups that rely on these annotations.

**Figure 3:**
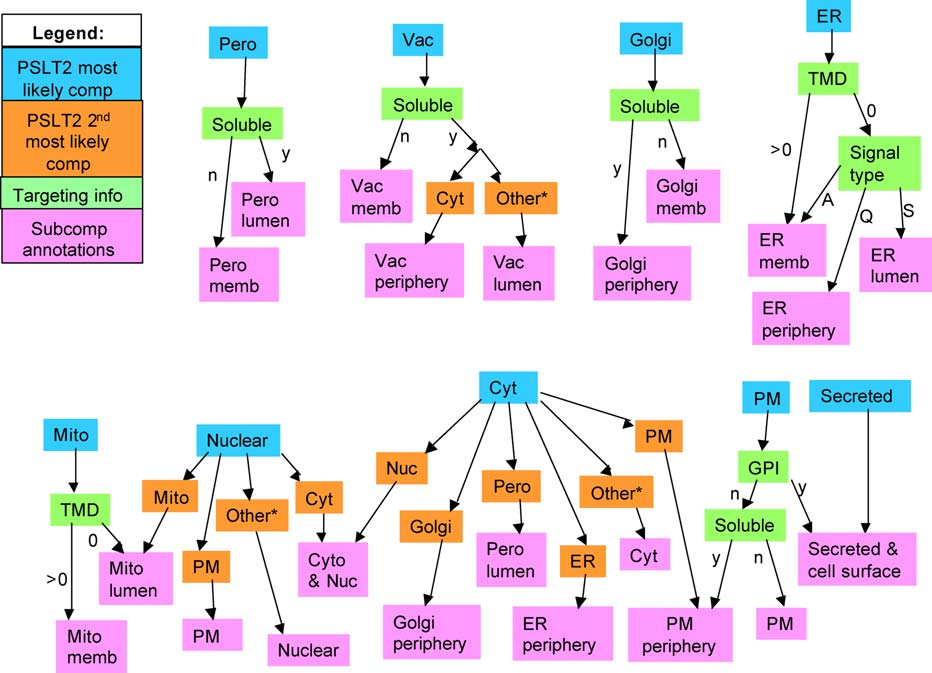
Sub-Compartmental Prediction Scheme Proteins are predicted to be localized in one specific sub-compartment by first considering the most likely PSLT2 compartment (blue boxes). Further decisions depend on the PSLT2 second most likely compartment prediction (orange boxes) and targeting information (green boxes). Once all information has been analyzed, the protein is predicted to be in one of 18 sub-compartments (pink boxes). When the second most likely sub-compartments are considered, the default prediction is shown with a star (this branch of the tree is used in particular when proteins have no second most likely compartment as predicted by PSLT2). Pero, peroxisome; Vac, vacuole; Cyt, cytosolic; memb, membrane; TMD, number of transmembrane domains in protein; ER, endoplasmic reticulum; GPI, presence of GPI anchor; Nuc, nuclear; PM, plasma membrane; Mito, mitochondria; S, signal peptide; A, signal anchor; Q, neither signal peptide nor signal anchor.

As illustrated in a previous section, the multi-compartmental prediction capacity of PSLT2 allows us to identify some groups of proteins that localize to the boundary of organelles. This is an interesting feature that can be exploited to refine protein subcellular localization predictions and, in particular, to distinguish between lumenal organellar proteins and cytosolic proteins peripherally associated with the organelle. We decided to use the cell-wide PSLT2 predictions in an attempt to annotate with greater detail the yeast proteome. We classify yeast proteins into 18 sub-compartments using PSLT2 localization predictions and targeting motif information (which is necessary because not all PSLT2 predictions allow us to distinguish between different sub-compartments; see Materials and Methods). The classification scheme used (shown in Figure 3) is a decision tree that was generated using the C4.5 software [28] and a manually curated training set consisting of 1,167 yeast proteins. We chose to use decision trees to perform such an analysis because of the nature of the data (the data are complete, and the input variables likely highly influence the output variables). In such situations, decision trees can encode the relationships between the variables with few parameters, which can result in more accurate classification [15]. Furthermore, decision trees provide a classification scheme from which it is easy to identify the rules used for the prediction. Decision trees have already been used to predict subcellular localization [29].

The training set was constructed using high-quality annotations in UniProt [24] and information in the literature. Full manual curation was necessary because few proteins are annotated with such detailed information. As shown in Figure 3, to predict sub-compartmental localization, the learnt decision tree first considers the most likely compartment as predicted by PSLT2. When this information is not sufficient to predict sub-compartmental localization, the decision tree next considers either the second most likely compartment as predicted by PSLT2 (if such a prediction exists), or other protein features, such as the number of transmembrane domains or the presence of GPI anchors. In some rare cases, further information is required, such as the presence of signal peptides. We originally also used mitochondrial targeting peptide predictions as an attribute considered by the decision tree software, but this resulted in a larger tree with no gain in accuracy. We chose to model the extracellular group of yeast proteins as consisting of both secreted and cell surface proteins. In the original PSLT2 training set, several proteins annotated as secreted are in fact soluble proteins anchored on the cell surface (yeast has few truly secreted proteins that do not remain in the periphery of the plasma membrane).

The accuracy of the predictor is measured to be 83% using a 10-fold cross-validation test. We used this decision tree predictor to annotate all yeast proteins with sub-compartmental information. The sub-compartmental training set and predictions are available in Table S2. Our new predictions have substantially increased the number of yeast proteins annotated in most of these sub-compartments (as shown in Table S3). For example, five proteins were previously annotated as being in the ER periphery. Our decision tree predictor brings this number up to 152. Such sub-compartmental predictions should be useful to the biology research community, especially in the case of proteins with no previous sub-compartmental annotations.

We further assessed the quality of our sub-compartmental predictions using a specific example that involves many proteins known to localize to sub-compartments: the UPR (unfolded protein response) and ERAD (ER-associated degradation) pathways related to quality control in the ER. Using a literature search, we determined the sub-compartmental localization of 30 proteins involved in these pathways. Our sub-compartmental predictions agree remarkably well with the experimentally determined localizations of these proteins (see Table S4).

### Localizome–Interactome Maps of the Secretory Pathway

As mentioned previously, PSLT2 achieves a good prediction accuracy for proteins in the secretory pathway, which is often not the case for most available localization predictors. In general, proteins in organelles of the secretory pathway and the plasma membrane are less well represented in large datasets generated by high-throughput strategies, probably because they are not amenable to some of the high-throughput experimental methods such as yeast-two hybrid and mass spectrometry. Many are membrane or membrane associated or require chemical environments that are different from those in which most proteins function—for example, the lumen of the ER. Our sub-compartment localization predictions provide a very useful new tool to investigate these proteins.

**Figure 4:**
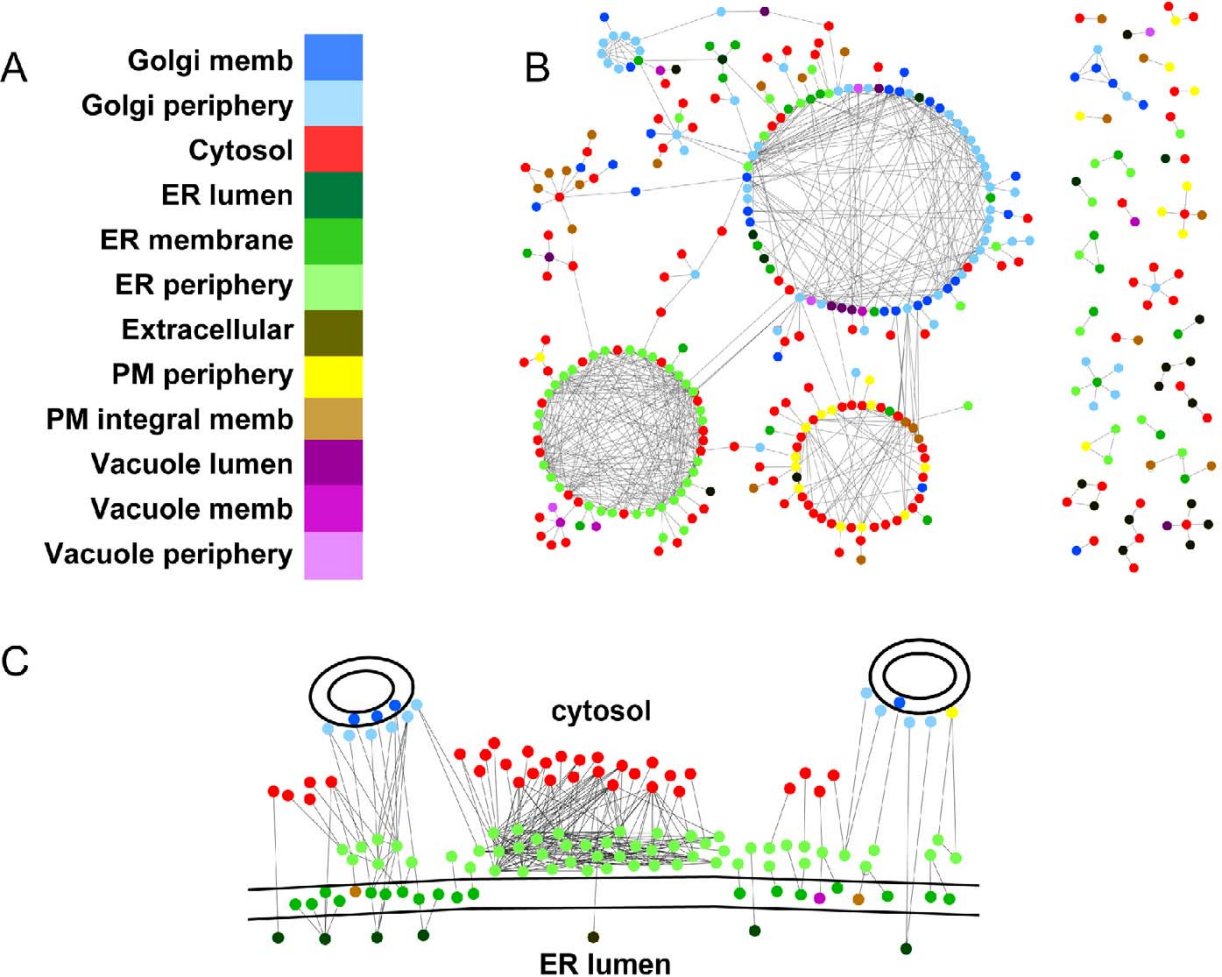
Localizome–Interactomes The protein–protein interaction maps for all proteins in the secretory pathway (B) or all proteins in the ER (C). Proteins are depicted as circles coloured according to their predicted sub-compartmental localization, as specified in the legend in (A). Interactions are shown as lines between proteins.

In Figure 4, we present the first (to our knowledge) such model of interaction–localization networks focused on the secretory pathway. To generate these networks, we use only the CORE protein–protein interactions available from the Database of Interacting Proteins [18], since these interactions are considered highly validated. The localization–interaction maps are generated by selecting all protein pairs annotated as being either in a secretory pathway sub-compartment or cytosolic and that are involved in protein–protein interactions annotated in the CORE dataset of the DIP. Figure 4B shows the localizome–interactome of the full secretory pathway. Visual inspection of this map suggests that large groups of co-localized proteins interact together and that protein sub-compartment annotations and protein–protein interaction data are generally consistent. For a large group of ER periphery proteins (lower left group, Figure 4B), interactions are almost exclusively between members of this group or with cytosolic proteins. Many peripheral Golgi and membrane proteins interact amongst themselves and with some cytosolic, peripheral ER, ER membrane, and vacuolar proteins (large middle group). And several cytosolic proteins interact with plasma membrane periphery proteins, plasma membrane integral membrane proteins, and vacuolar proteins (lower middle group). In general, interactions involve proteins that are localized in the same or adjacent sub-compartments, and there is extensive cross-talk between cytosolic proteins and proteins at the periphery of organelles.

Figure 4C shows the localizome–interactome of all proteins predicted to be in one of the three ER sub-compartments and their interactors (according to DIP). Once again, a large group of ER periphery proteins interact amongst themselves, and with cytosolic proteins. Several interactions involve Golgi periphery and ER periphery or membrane proteins. Literature searches indicate that many of these Golgi periphery proteins are known to localize to COPII vesicles that traffic between the ER and the Golgi, and could thus interact with proteins in the periphery and the membrane of the ER. The lower left-most area in Figure 4C also shows some ER membrane proteins interacting with ER lumenal and cytosolic proteins. These proteins are involved in protein translocation across the ER membrane, vesicle formation between the ER and Golgi, N-linked glycosylation, phospholipid metabolism, and oxidative folding, all known to be important functions of the ER. Dispersed throughout the network, a few ER membrane and lumenal proteins interact with cargo proteins that likely transit through the ER, such as plasma membrane, vacuole membrane, and extracellular proteins.

## Discussion

We introduce here PSLT2, a subcellular localization predictor for yeast proteins that achieves high prediction accuracies for most compartments considered, including secretory pathway organelles. PSLT2 can identify proteins that are potentially present in more than one compartment and has been used to predict the localization of all yeast proteins. PSLT2 combines a methodology initiated for PSLT [5] (mostly in the *motif* module) with additional information that is widely available for yeast. This is mainly interaction data and the presence of various targeting signals. Integration results in a more complete model to predict localization. As large-scale protein–protein interaction maps are being generated for a growing number of organisms [30–33], protein interaction data will certainly become an important type of evidence for the prediction of localization. We show here that the integration of these different types of data in an appropriate framework greatly increases the prediction accuracy and coverage.

A comparison between our predictions and annotations from two large high-throughput datasets shows that disagreements between these methods generally occur in proteins that transit through or localize in organelles of the secretory pathway. While it is probable that some of these proteins are mislocalized in the procedures for high-throughput tagging, possibly due to masking or disruption of targeting signals, it is certainly also the case that some PSLT2 predictions are incorrect. Examples of proteins that are poorly predicted by PSLT2 include enzyme families of which two isozymes are known to localize to separate compartments. We are currently investigating other types of additional information that could be used by our predictor to increase its accuracy in such cases. In general, high-throughput experimental methods and bioinformatic predictors should be used in parallel, and disagreements between these two types of methods can be viewed as indicators of cases where further experimental validation using independent methods is required.

### Sub-Compartmental Localization

Using PSLT2 predictions and the presence of specific targeting signals, we have investigated how localization annotations can be refined to distinguish between lumenal organellar and organellar periphery annotations. Such refined predictions are of particular interest because they offer a new picture of the protein composition of the surface of organelles, which in turn can help understand mechanisms of communication between different cellular compartments. In addition, these predictions will likely also be of particular use in the validation of high-throughput protein–protein interaction datasets, which are currently often validated using conventional protein localization annotations [34] that do not distinguish between lumenal and peripheral organellar proteins and use the “cytoplasmic” annotation rather the “cytosolic” annotation, which is more specific.

We have used our sub-compartmental predictions to initiate the investigation of the localizome–interactome of the secretory pathway. The models that can be derived from this analysis provide a novel global view of interactions and complexes in these organelles, as well as of cross-talk between these organelles at a level that has never, to our knowledge, been investigated previously.

The large-scale study of protein–protein interactions in the secretory pathway has lagged behind such efforts in other parts of the cell. This will soon change as several methods are currently being developed that can specifically overcome the challenges offered by these organelles [35,36]. Our sub-compartmental localization predictions can be useful in the choice and technical manipulation of protein targets to study using these novel protein–protein interaction discovery platforms. The data generated by these novel strategies, in combination with methods such as ours, will provide a better global understanding of these organelles.

## Materials and Methods

### Components of the PSLT2 Bayesian network.

The PSLT2 predictor is composed of three independent modules that can predict localization on their own or in combination, as shown in Figure 1.

The *motif* module predicts localization based on the co-occurrence of InterPro motifs [16] in proteins as previously described [5]. Briefly, the likelihood of localization to all compartments considered is calculated and stored in an XML file for all combinations of motifs present in all proteins in the training set, using a dynamic programming algorithm. During this training phase of the algorithm, several thousands of motif combinations are considered. At the completion of this phase, given an unknown protein and the InterPro motifs it contains, the likelihood of localization to all compartments can be found by looking up this information in the motif-likelihood XML file. It should be noted that the motif module is a Bayesian Network structural learning problem that we solve by looking at all combinations of motifs present in proteins in the training set using dynamic programming. This approach is feasible with the number of proteins and motifs considered here but might become infeasible as more motifs are added to InterPro and more proteins are considered. In such a case, other approaches, including structural expectation-maximization (EM) could be used [15].

The *targeting* module is a full Bayesian network that considers all combinations of the presence of signal peptides, mitochondrial targeting peptides, GPI anchors, and the number of transmembrane domains in proteins to predict localization. The presence of these signal/domains is predicted respectively by SignalP [9], TargetP [10], DGPI (http://129.194.185.165/dgpi/index_en.html) and TMHMM [17]. The localization likelihood to all compartments given all these targeting signals is evaluated by using Bayes rule:


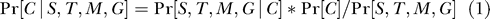


where S indicates the presence of signal peptides; M, the presence of mitochondrial targeting motifs; T, the number of transmembrane domains; and G, the presence of GPI anchors. The compartment prior Pr[C], the targeting signal prior Pr[S,T,M,G], and the targeting signal posterior Pr[S,T,M,G|C] are each evaluated by counting proteins in the training set. The information used by the *targeting* module is available for all proteins regardless of whether they have been previously characterized.

The *interaction* module predicts localization likelihoods to all compartments considered, given the localization of known interacting partners. This module is trained using the CORE DIP dataset [18], which represents the most reliable interactions within the full DIP dataset. For each protein X, we create a vector representing the three compartments that contain the most protein interacting partners for protein X according to the CORE DIP dataset. Then the localization likelihood to all compartments considered can be evaluated for all vectors present in the training set using Bayes rule.

All three modules output the likelihood of localization to all compartments considered and as such can predict localization on their own. When used in combination, their localization likelihoods are combined in a naïve Bayesian network according to:


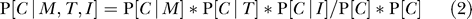


where C is the cellular compartment, M and T represent the motifs and targeting signals present in the protein, respectively, and I represents the localization vector of the interaction partners of the protein whose localization is being predicted.

To avoid a situation where at least one of the three modules predicts a localization likelihood of zero for a given compartment while the other module(s) predict a non-zero localization likelihood for the compartment (which would result in the full predictor outputting a likelihood of zero for that compartment), we add pseudo-counts to the localization likelihoods of all three modules. These pseudo-counts are very small and reflect the underlying probability of presence in a compartment (here, we use one-tenth of the compartment prior P[C] for the pseudo-counts).

As mentioned previously, predictions for all of the targeting motifs used by the *targeting* modules are available for all yeast proteins. Such is not the case for the *motif* and *interaction* modules. Indeed, not all proteins contain InterPro motifs or interaction partners. We say a protein is uninformative if it lacks both motif and interaction information. Otherwise, we say it is informative. It should be noted that the use of pseudo-counts for all three modules is equivalent to eliminating the module (either *motif* or *interaction*) for which no informative information is available.

The compartment priors P[C] are estimated as described previously [5].

### PSLT2 datasets.

The PSLT2 Bayesian network is trained using information in UniProt [24] and curated from the literature. UniProt is a curated database of protein sequences that provides a high level of annotation, including subcellular localization information. Localization annotations were kept if they clearly indicated which compartment(s) the protein is localized to. No annotations described as “possible,” “potential,” “probable,” or “by similarity” were kept. The PSLT2 training set contains localization information for 1521 *S. cerevisae* proteins localized to nine different compartments (several are localized to more than one compartment).

Two high-throughput localization datasets were also used in this study: the YeastGFP dataset [2] and the TRIPLES dataset [1]. The YeastGFP dataset consists of 4,156 yeast proteins whose localization was established by visualization of chromosomally tagged GFP fusion proteins. The TRIPLES dataset was derived by immunolocalization of 2,744 tagged yeast proteins.

### Comparison between datasets.

The annotations of three datasets (PSLT2, YeastGFP, and TRIPLES) were compared by calculating the Pearson correlation for the probability of localization between all compartment pairs for each pair of datasets. More specifically, the vector of probabilities of localization to all compartments for all proteins in a dataset is compared to an equivalent vector for all proteins in a second dataset.

### Sub-compartment prediction.

We predict sub-compartmental localization using the C4.5 software for the creation of decision trees [28]. The sub-compartmental annotation mainly involves distinguishing between lumenal, membrane, and peripherally associated proteins for most organelles. The attributes considered by the predictor are the PSLT2 predictions, the number of transmembrane domains, and some targeting signals. This new predictor is trained using a fully manually curated dataset that incorporates high-quality annotations from UniProt [24] and information available in the literature. This dataset contains subcompartmental annotation for 1,167 *S. cerevisiae* proteins (these annotations are available in Table S2, column “Sub-compartmental localization annotation from UniProt or literature”). The manual construction of such a dataset is necessary because few proteins are annotated with such precise localization information. Table S2 also contains all the training information used during the prediction (columns “Signal type,” “Topology,” “Targeting type,” and “GPI anchor”). Proteins that are predicted to contain only one transmembrane domain by TMHMM [17] that overlaps with the signal peptide predicted by SignalP [9] are considered to be soluble.

The predictor is created using the iterative mode of C4.5 during which the software sequentially creates more accurate decision trees by using more training examples until no further improvement is obtained. One hundred different trees were created (almost identical to one another in this case) and the most accurate tree is chosen as the decision tree that provides the classification scheme for our sub-compartmental predictions.

### Localizome–interactome maps.

The localizome-interactome maps are generated using Cytoscape [37]. The datasets are created by selecting all protein pairs annotated as being in a secretory pathway sub-compartment or cytosolic and that are involved in protein–protein interactions annotated in the CORE dataset of the Database of Interacting Proteins [18]. The nodes are coloured according to their sub-compartmental predictions.

## Supporting Information

Table S1 PSLT2 Predictions (511 KB XLS)

Table S2 Sub-Compartmental Localization Dataset (876 KB XLS)

Table S3 Number of Proteins Previously Annotated and Newly Predicted in Each Sub-Compartment Considered (26 KB DOC)

Table S4 Sub-Compartmental Analysis of the UPR and ERAD Pathway Components (54 KB PDF)

## Abbreviations

ER: endoplasmic reticulum
GPI: glycosyl-phosphatidylinositol
