# Supplementary material for: Refining Protein Subcellular Localization
Source: PLoS Comput Biol. 2005 Nov 25;1(6):e66. doi: 10.1371/journal.pcbi.0010066 (PMC1289393; doi:10.1371/journal.pcbi.0010066)
Supplement: Table S3 — (26 KB DOC) [file pcbi.0010066.st003.doc]

| **Sub-compartments** | **Number of proteins previously annotated** | **Number of proteins predicted in this study** |
| --- | --- | --- |
| Cytosolic | 171 | 651 |
| Cytosolic and nuclear | 114 | 2309 |
| ER lumen | 8 | 47 |
| ER membrane | 41 | 447 |
| ER periphery | 5 | 152 |
| Golgi membrane | 24 | 65 |
| Golgi periphery | 18 | 107 |
| Mitochondrion lumen | 180 | 682 |
| Mitochondrion membrane | 49 | 162 |
| Mitochondrion periphery | 9 | 0 |
| Nucleus membrane | 3 | 0 |
| Nuclear (soluble) | 429 | 1015 |
| Peroxisome lumen | 13 | 50 |
| Peroxisome membrane | 8 | 11 |
| Plasma membrane | 21 | 315 |
| Plasma membrane periphery (cytosolic side) | 17 | 75 |
| Plasma membrane periphery (outside the cell) | 24 | 216 |
| Vacuole lumen | 6 | 21 |
| Vacuole membrane | 19 | 34 |
| Vacuole periphery | 6 | 4 |

**Supplementary table III**: Number of proteins previously annotated and newly predicted in each sub-compartment considered.
