# Supplementary material for: Refining Protein Subcellular Localization
Source: PLoS Comput Biol. 2005 Nov 25;1(6):e66. doi: 10.1371/journal.pcbi.0010066 (PMC1289393; doi:10.1371/journal.pcbi.0010066)
Supplement: Table S4 — (54 KB PDF) [file pcbi.0010066.st004.pdf]

Sub-compartmental analysis of the UPR and ERAD pathway components

Common Name	SGD Systematic Name	Literature derived localization annotation	PSLT2 prediction	Sub-compartmental prediction	GO cellular compartment	Function
Scj1	YMR214W	ER lumen	ER	ER membrane	ER lumen	One of several homologs of bacterial chaperone DnaJ, located in the ER lumen where it cooperates with Kar2p to mediate maturation of proteins
Ssa1	YAL005C	ER periphery	Cytosol	cytosolic	Nuc; cell wall; cytoplasm	ATPase; hsp70; complex with Ydj1p
Ydj1	YNL064C	ER periphery	Cytosol	cytosolic and nuclear	Cytosol	Protein chaperone involved in regulation of the HSP90 and HSP70 functions; involved in protein translocation across membranes; member of the DnaJ family
Kar2	YJL034W	ER lumen	ER	ER lumen	ER lumen	ATPase involved in protein import into the ER, also acts as a chaperone to mediate protein folding in the ER and may play a role in ER export of soluble proteins; regulates the unfolded protein response via interaction with Ire1p
Hlj1	YMR161W	ER membrane	ER	ER membrane	ER membrane	Co-chaperone for Hsp40p; with its homolog Hdj1p promotes ERAD; similar to DnaJ
Cne1	YAL058W	ER membrane	ER	ER membrane	int. ER	ER chaperone involved in the folding and quality control of glycoproteins
Jem1	YJL073W	ER lumen	ER	ER lumen	around N-ER membrane	DnaJ-like chaperone required for nuclear membrane fusion during matin
Lhs1	YKL073W	ER lumen	ER	ER lumen	ER lumen	Molecular chaperone LERP, involved in polypeptide translocation and folding; member of the Hsp70 family; regulated by UPR
Npl4	YBR170C	nuclear - ER periphery	ER	ER periphery	nuclear-ER periphery	Endoplasmic reticulum and nuclear membrane protein, forms a complex with Cdc48p and Ufd1p that recognizes ubiquitinated proteins in the endoplasmic reticulum and delivers them to the proteasome for degradation
Cdc48	YDL126C	ER periphery	ER	ER periphery	Cyt; ER; Nucleus; microsome	participates in retrotranslocation of ubiquitinated proteins from the ER into the cytosol for degradation by the proteasome
Ufd1	YGR048W	ER periphery	PM	plasma membrane periphery	ER	Protein that interacts with Cdc48p and Npl4p, involved in recognition of polyubiquitinated proteins and their presentation to the 26S proteasome for degradation; involved in transporting proteins from the ER to the cytosol
Sec63	YOR254C	ER membrane	ER	ER membrane	ER membrane	Essential subunit of Sec63 complex (Sec63p, Sec62p, Sec66p and Sec72p); with Sec61 complex, Kar2p/BiP and Lhs1p forms a channel competent for SRP-dependent and post-translational SRP-independent protein targeting and import into the ER
Ssm4	YIL030C	ER periphery	ER	ER membrane	int. ER; int Nuc	Ubiquitin-protein ligase of the ER/nuclear envelope
Hrd1	YOL013C	ER membrane	ER	ER membrane	ER membrane	Ubiquitin-protein ligase required for ERAD; genetically linked to UPR; regulated through association with Hrd3p

Rpn1	YHR027C	ER periphery	ER	ER periphery	cyto;nuc;er;proteasome	Non-ATPase base subunit of the 19S regulatory particle of the 26S proteasome; may participate in the recognition of several ligands of the proteasome; contains a leucine-rich repeat (LRR) domain, a site for protein?protein interactions
Mnl1	YHR204W	ER lumen	ER	ER lumen	ER	
Cue1	YMR264W	ER membrane	Cytosol	mitochondria lumen	ER membrane; mito.	recruits the ubiquitin-conjugating enzyme Ubc7p to the ER where it functions in protein degradation
Ubc5	YDR059C	ER periphery	Cytosol	cytosolic and nuclear	Proteasome	Ubiquitin-conjugating enzyme that mediates selective degradation of short-lived and abnormal proteins, central component of the cellular stress response; expression is heat inducible
Ubc6	YER100W	ER membrane	Cytosol	cytosolic	ER membrane/Cytosolic	Ubiquitin-conjugating enzyme involved in ER-associated protein degradation; located at the cytosolic side of the ER membrane; tail region contains a transmembrane segment at the C-terminus; substrate of the ubiquitin-proteasome pathway
Qri8/Ubc7	YMR022W	ER periphery	ER	ER periphery	ER periphery	Ubiquitin conjugating enzyme, involved in the ER-associated protein degradation pathway; requires Cue1p for recruitment to the ER membrane; proposed to be involved in chromatin assembly
Ptc2	YER089C	ER periphery	Cytosol	cytosolic	Cytosol; Nuclear	Type 2C protein phosphatase; dephosphorylates Hog1p to limit maximal osmostress induced kinase activity; dephosphorylates Ire1p to downregulate the unfolded protein response; dephosphorylates Cdc28p; role in DNA checkpoint inactivation
Hac1	YFL031W	nuclear - ER periphery	Nuclear	nuclear	Nuclear	bZIP transcription factor (ATF/CREB1 homolog) that regulates the unfolded protein response, via UPRE binding, and membrane biogenesis; ER stress-induced splicing pathway utilizing Ire1p, Trl1p and Ada5p facilitates efficient Hac1p synthesis
Ire1	YHR079C	ER membrane	ER	cytosolic and nuclear	nER membrane	Serine-threonine kinase and endoribonuclease; transmembrane protein that initiates the unfolded protein response signal by regulating synthesis of Hac1p through HAC1 mRNA splicing
Orm2	YLR350W	ER membrane	ER	plasma membrane	ER membrane (tail)	Evolutionarily conserved protein with similarity to Orm1p, required for resistance to agents that induce the unfolded protein response; human ortholog is located in the endoplasmic reticulum
Gcn4	YEL009C	nuclear - ER periphery	Nuclear	nuclear	Nuclear	Transcriptional activator of amino acid biosynthetic genes in response to amino acid starvation
Spt20	YOL148C	nuclear	Nuclear	nuclear	Nuclear	Subunit of the SAGA transcriptional regulatory complex, involved in maintaining the integrity of the complex
Erv46	YAL042W	ER membrane	ER	ER membrane	ER membrane; golgi membrane; COP-II vesicle	forms a complex with Erv41p; involved in membrane fusion stage of transport

Pdi1	YCL043C	ER lumen	ER	ER lumen	ER lumen	multifunctional protein resident in the endoplasmic reticulum lumen, essential for the formation of disulfide bonds in secretory and cell-surface proteins, unscrambles non-native disulfide bonds
Rlg1/Trl1	YJL087C	nuclear - ER periphery	Cytosol	cytosol and nuclear	Nuclear inner membrane	tRNA ligase, required for tRNA splicing; composed of three essential domains containing the phosphodiesterase, polynucleotide kinase, and ligase activities required for ligation; localized at the inner membrane of the nuclear envelope
